# Spent Material Extractives from Hemp Hydrodistillation as an Underexplored Source of Antimicrobial Cannabinoids

**DOI:** 10.3390/antibiotics13060485

**Published:** 2024-05-24

**Authors:** Simon Vlad Luca, Krzysztof Wojtanowski, Izabela Korona-Głowniak, Krystyna Skalicka-Woźniak, Mirjana Minceva, Adriana Trifan

**Affiliations:** 1 Biothermodynamics, TUM School of Life Sciences, Technical University of Munich, 85354 Freising, Germany; mirjana.minceva@tum.de; 2 Department of Pharmacognosy with Medicinal Plant Unit, Medical University of Lublin, 20-093 Lublin, Poland; krzysztof.wojtanowski@umlub.pl; 3 Department of Pharmaceutical Microbiology, Faculty of Pharmacy, Medical University of Lublin, 20-093 Lublin, Poland; iza.glowniak@umlub.pl; 4 Department of Natural Products Chemistry, Medical University of Lublin, 20-093 Lublin, Poland; kskalicka@pharmacognosy.home.pl; 5 Department of Pharmacognosy-Phytotherapy, Faculty of Pharmacy, “Grigore T. Popa” University of Medicine and Pharmacy Iasi, 700115 Iasi, Romania

**Keywords:** *Cannabis sativa* L., cannabidiol, CBD, essential oil, by-products, terpenes, antimicrobial, fatty acids

## Abstract

Hemp (*Cannabis sativa* L.) has been used for millennia as a rich source of food and fibers, whereas hemp flowers have only recently gained an increased market interest due to the presence of cannabinoids and volatile terpenes. Currently, the hemp flower processing industry predominantly focuses on either cannabinoid or terpene extraction. In an attempt to maximize the valorization of hemp flowers, the current study aimed to evaluate the phytochemical composition and antimicrobial properties of several extracts obtained from post-distillation by-products (e.g., spent material, residual distillation water) in comparison to the essential oil and total extract obtained from unprocessed hemp flowers. A terpene analysis of the essential oil revealed 14 monoterpenes and 35 sesquiterpenes. The cannabinoid profiling of extracts showed seven acidic precursors and 14 neutral derivatives, with cannabidiol (CBD) reaching the highest concentration (up to 16 wt.%) in the spent material extract. The antimicrobial assessment of hemp EO, cannabinoid-containing extracts, and single compounds (i.e., CBD, cannabigerol, cannabinol, and cannabichromene) against a panel of 20 microbial strains demonstrated significant inhibitory activities against Gram-positive bacteria, *Helicobacter pylori*, and *Trichophyton* species. In conclusion, this work suggests promising opportunities to use cannabinoid-rich materials from hemp flower processing in functional foods, cosmetics, and pharmaceuticals with antimicrobial properties.

## 1. Introduction

Hemp (*Cannabis sativa* L., Cannabaceae) has served for over 6000 years as a versatile resource for food, fibers, oils, medicines, and recreational or religious activities [1]. Throughout the Middle Ages, hemp became a vital fiber crop for the production of textiles and ropes [2]. However, following the discovery of the central nervous system (CNS)-intoxicating effects of Δ^9^-tetrahydrocannabinol (Δ^9^-THC), an important constituent in its flowers, hemp cultivation has decreased over the 20th century [3]. Nevertheless, at the beginning of the 2000s, controlled genotypes (containing less than 0.2–0.3% Δ^9^-THC) were re-authorized to be marketed for agricultural purposes in the European Union [2]. Consequently, more than 60 cultivars have been registered since [4]. Thanks to the wide application of its derivatives, hemp cultivation has ramped up in the last few decades; for instance, fibers and hurds are used for the textile, paper, construction, and automotive industries; biomass is used for the production of biofuels (biodiesel, bioethanol, biogas); and lastly, the oils derived from seeds are regarded as food, cosmetic ingredients, or animal feed [2,3]. Nevertheless, from the global perspective of a sustainable circular economy, more attention has been recently paid to utilizing agro-industrial waste within the hemp production chain, with particular emphasis on the flowers. Hemp flowers/inflorescences are valuable sources of high-adding value compounds, in particular cannabinoids and volatile terpenes.

Cannabinoids are a class of terpeno-phenolics encompassing more than 150 members. These constituents are biosynthesized in the secretory cells of glandular trichomes, primarily concentrated in unfertilized female flowers [5]. Unlike Δ^9^-THC, cannabidiol (CBD) lacks CNS-intoxicating properties and has antiemetic, anti-inflammatory, antioxidant, antimicrobial, and neuromodulatory effects. CBD is currently under clinical evaluation for the treatment of 26 medical conditions and has already been granted orphan drug status for 11 other diseases [6]. Additionally, the Food and Drug Administration and the European Medicines Agency have approved Epidiolex^®^/Epidyolex^®^ as the first CBD-based medication for adjunctive therapy in treating seizures linked to Dravet and Lennox-Gastaut syndromes [7]. As compared to CBD, other cannabinoids, such as cannabigerol (CBG), cannabidivarin (CBDV), cannabinol (CBN), or cannabichromene (CBC), are regarded as minor constituents since they are present in significantly lower amounts in hemp flowers. Preliminary pharmacological investigations have shown promising therapeutic attributes for some of the minor cannabinoids, such as antimicrobial, anti-inflammatory, neuroprotective, anticonvulsant, antiemetic, anti-psoriatic, anticancer, anti-melanogenic, or antidepressant effects [6,8,9,10,11].

Besides cannabinoids, volatile terpenes are another class of constituents secreted in the trichomes of female hemp flowers [12]. More than 90 terpenes have been reported in hemp, formally grouped into monoterpenes (e.g., α-pinene, β-pinene, β-myrcene, limonene) and sesquiterpenes (e.g., β-caryophyllene, α-humulene, β-caryophyllene oxide) [13]. These compounds generally confer a mild, light floral fragrance to the hemp flowers, allowing them to be used in perfumery or cosmetics. In addition, hemp terpenes were shown to exert interesting pharmacological properties, such as insecticidal, neuromodulatory, antimicrobial, anticancer, or anti-inflammatory effects [14,15,16]. Thus, niche products, such as hemp essential oils (EO), usually isolated by steam distillation or hydrodistillation, are currently available on the market [15]. However, in light of the low EO yields achieved from hemp flowers, the residual (spent) plant material remaining at the end of steam or hydrodistillation is considerably high (up to 99.7 wt.%). In addition, a diverse array of other post-distillation by-products are generated, such as aqueous condensates (hydrolates, hydrosols) and distillation waters (residual waters, leachates). Since there is no information concerning the further use of these materials, it can be assumed that they are simply regarded as waste and disposed of in the environment, similar to other waste stream products from the aroma and EO industry [17].

In an attempt to find uses for the above-mentioned by-products that could arise from hemp flower hydrodistillation, this study evaluated the phytochemical profile and antimicrobial activity of several post-distillation extracts in comparison to the essential oil and total extract obtained from unprocessed hemp flowers. Besides our ongoing multi-team research efforts to find novel naturally derived chemotherapeutic agents for further use in treating human infections, our interest in screening hemp’s antibacterial and antifungal properties has also been motivated by several preliminary reports that scarcely explored the inhibitory properties of hemp extracts and purified cannabinoids against Gram-positive bacteria, yeasts, and certain Gram-negative bacteria [18,19,20,21,22,23,24]. Thus, this work aimed to obtain a hydrodistilled hemp flower essential oil and use the remaining by-products (e.g., spent material, residual distillation water) as an underexplored source of cannabinoids for a further assessment of their antibacterial and antifungal properties.

## 2. Results and Discussion

In this study, the essential oil from hemp flowers obtained by hydrodistillation (HEO) was analyzed by gas chromatography coupled with mass spectrometry (GC–MS). In parallel, the residual water used for hydrodistillation was concentrated (HWE), whereas the processed hemp flowers (spent material after hydrodistillation) were extracted with hexane (HSE). For comparison purposes, the unprocessed hemp flowers were also extracted under the same conditions (HTE) (Table 1). The fatty acid composition of the solvent extracts was analyzed by GC–MS, whereas the cannabinoid profile was assessed qualitatively by liquid chromatography hyphenated with high-resolution tandem mass spectrometry (LC-HRMS/MS) and quantitatively by liquid chromatography coupled with diode array detection (LC-DAD). Subsequently, the antimicrobial activity of hemp essential oil, cannabinoid-containing extracts, and individual cannabinoids (i.e., CBD, CBG, CBN, and CBC) was tested against a panel of 20 microbial strains.

### 2.1. Terpene Profile of Hemp Essential Oil

**Figure 1:**
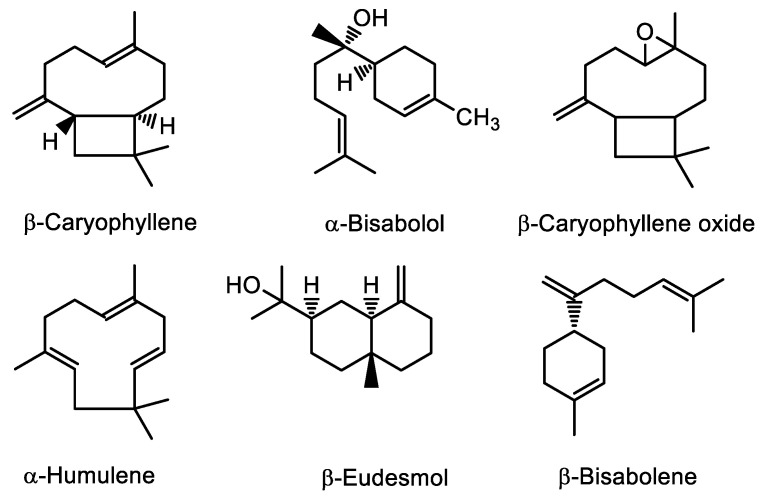
Structures of main terpenes identified in the hemp essential oil.

According to the obtained terpene profile data (Table 2), sesquiterpenes in HEO accounted for 85.94 ± 0.63% of the total peaks, with almost an equal distribution between oxygenated and non-oxygenated (hydrocarbon) sesquiterpenes. (*E*)-*β*-Caryophyllene (Figure 1) was the dominant compound in HEO (17.17 ± 0.24%), followed by β-caryophyllene oxide (8.22 ± 0.13%), α-bisabolol (8.20 ± 0.12%), α-humulene (6.12 ± 0.53%), *β*-eudesmol (5.79 ± 0.05%), and *β*-bisabolene (5.14 ± 0.05%). From the group of monoterpenes, *α*-pinene and *β*-myrcene are worth mentioning, even though their relative amounts were only 2.80 ± 0.04% and 1.09 ± 0.02%, respectively (Table 2).

Our data are consistent with those reported in a previous work, when β-caryophyllene, α-humulene, β-caryophyllene oxide, and *β*-eudesmol were found in relative amounts of 16.20 ± 0.96%, 6.51 ± 0.45%, 6.39 ± 0.53%, and 5.20 ± 0.26%, respectively [25]. In another study, Pieracci et al. [26] showed that hemp EOs obtained from 10 different genotypes of *C. sativa* were characterized by a predominance of oxygenated sesquiterpenes ranging from 30.90 ± 7.02% to 60.90 ± 2.48%, with *β*-caryophyllene, *α*-humulene, *β*-caryophyllene oxide, and humulene epoxide as the typical constituents. Menghini et al. [27] reported the presence of *β*-caryophyllene (19.3%), α-humulene (8.3%), and *β*-caryophyllene oxide (4.3%) in an EO obtained from *C. sativa* cv. Futura 75; in addition, the same group of authors also noticed higher relative amounts of monoterpenes, such as α-pinene (14.9%), *β*-myrcene (11.8%), and *β*-pinene (3.8%), than in our study. Furthermore, Zheljazkow et al. [28] claimed that the concentrations of the main hemp terpenes in cultivated and wild hemp ascensions varied between 11.0–29.6% for β-caryophyllene, 4.4–11.9% for α-humulene, 0.2–31.2% for β-caryophyllene oxide, and 1.2–9.5% for humulene epoxide.

### 2.2. Cannabinoid Profile of Hemp Extracts

According to the extraction yields (Table 1), the processed plant material gave a yield of extraction higher than the unprocessed one. This could be related to the fact that the water used for the hydrodistillation extracted many polar components from the initial hemp flowers. Therefore, the same dried mass of processed hemp flowers contains more hydrophobic components (easily extracted with hexane) than the same mass of the initial hemp flowers [17]. The cannabinoid profile of HWE, HSE, and HTE was assessed by LC—HRMS/MS. Due to the lack of standards, the identity of the cannabinoids was tentatively annotated according to previously developed methodologies [29,30,31,32]. For instance, the discrimination between CBD-, THC-, and CBC-type acidic cannabinoids was already presented by Piccolella et al. [32] for 20 different compounds identified in hemp pollen samples from Italy. Borille et al. [30] proposed the annotation of around 70 different cannabinoids from 68 Brazilian samples of *Cannabis* spp. (leaves, stems, and flowers). In addition, Berman et al. [29] performed the comprehensive metabolic profiling of 36 samples of *Cannabis* plants from Israel, identifying 94 cannabinoids belonging to 10 distinct subclasses. Barhdadi et al. [31] identified and quantified 17 cannabinoids in 20 CBD-based e-cigarette liquids from the Belgium market.

**Figure 2:**
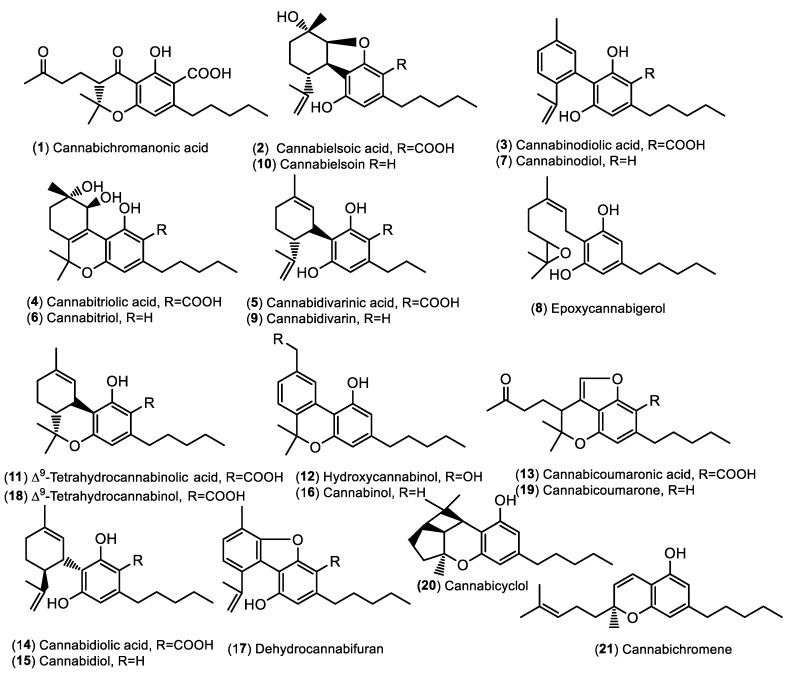
Chemical structures of cannabinoids identified in the hemp extracts.

According to the above-mentioned literature records and standard injections, 21 compounds were putatively identified in HWE, HSE, and HTE (Table 3, Figure 2). Based on the obtained data (Table 3), there were no essential qualitative differences between the extracts, i.e., most peaks were detected in all samples. The annotated cannabinoids can be grouped into two categories: acidic and neutral cannabinoids. With few exceptions, the acidic precursors and the corresponding decarboxylated derivatives were both present in the extracts; e.g., cannabielsoic acid (CBEA, **2**) + cannabielsoin (CBE, **10**); cannabinodiolic acid (CBNDA, **3**) + cannabinodiol (CBND, **7**); cannabitriolic acid (CBTA, **4**) + cannabitriol (CBT, **6**); cannabidivarinic acid (CBDVA, **5**) + CBDV (**9**); tetrahydrocannabinolic acid (Δ^9^-THCA, **11**) + Δ^9^-THC (**18**); cannabicoumaronic acid (**13**) + cannabicoumarone (**19**); cannabidiolic acid (CBDA, **14**) + CBD (**15**). On the other hand, the neutral form of cannabichromanonic acid (**1**) was not present, while the acidic precursors from epoxycannabigerol (**8**), hydroxycannabinol (**12**), CBN (**16**), dehydrocannabifuran (**17**), cannabicyclol (CBL, **20**), and CBC (**21**) were not identified.

### 2.3. Cannabinoid Composition of Hemp Extracts

The quantification of cannabinoids (also referred to as ‘cannabinoid potency’) is an important step in assessing the quality of a hemp flower-derived product. According to the results presented in Table 4, CBD was the major cannabinoid in all extracts (up to 15.93 ± 0.02 wt.%). Compared to HTE, HSE displayed CBD levels around two times higher, while the CBD concentration in the residual water extract HWE was ten times lower. This tendency could be correlated to the lipophilic character of the cannabinoids, conferring them a low extractability in water and high extractability in organic solvents. Interestingly, the acidic precursor CBDA was found in significant amounts in the unprocessed flower extracts HTE but in very low levels in the spent material extracts (Table 4). This could indicate a thermal conversion of CBDA to CBD during hydrodistillation due to exposing CBDA to a high temperature (~100 °C) for a long time (3 h). The remaining cannabinoids (e.g., CBDV, CBG, CBC, CBN, etc.) were found in concentrations between 0.01 to 0.55 wt.% (Table 4).

Our data are comparable with previous studies reporting on the cannabinoid concentration of various hemp extracts. For example, the concentrations of CBD, CBC, CBG, CBN, and CBL in methanol hemp flower extracts were 2.50 ± 0.10, 0.33 ± 0.12, 0.13 ± 0.01, 0.02 ± 0.00, and 0.02 ± 0.00 wt.%, respectively [33]. Muscara et al. [34] reported the presence of CBD, CBDA, cannabigerolic acid, CBN, and Δ^9^-THCA in amounts of 23.51 ± 0.05, 14.65 ± 0.01, 0.29 ± 0.01, 0.36 ± 0.01, 0.23 ± 0.01, and 0.03 ± 0.01 wt.%, respectively, in a hemp flower hexane extract.

### 2.4. Fatty Acid Composition of Hemp Extracts

Even though hemp flower extracts are known to contain lipids, their fatty acid composition is usually neglected. In all samples (Table 5), linoleic acid (C18:2) and linolenic acid (C18:3) were found in the highest amounts (up to 1.75 ± 0.07 wt.%). Significant concentrations of palmitic (C16:0) and arachidic acid (C20:1) were also noticed. Concerning its high polarity, the residual water extract HWE contained 15–50 times lower quantities of fatty acids than the solvent extracts. Generally, HSE displayed slightly higher values of fatty acids than HTE. This can be related to the fact that, during hydrodistillation, lipids are neither entrained with the volatile terpenes nor transferred into the residual water.

To our knowledge, there are no previous data concerning the fatty acid composition of hemp flower extracts. However, there are numerous similarities with the composition of hemp seeds. Kriese et al. [35] showed that linoleic acid (C18:2) and linolenic acid (C18:3) are the predominant fatty acids in the oils extracted from the seeds of 51 *C. sativa* genotypes. Their levels varied from 15.00–19.89 wt.% for linoleic acid and 5.15–8.24 wt.% for linolenic acid. Furthermore, numerous studies confirmed that linoleic acid (C18:2) is the major constituent of hempseed oils (up to 60% of the total fatty acids) [36,37,38,39].

### 2.5. Antimicrobial Activity of Hemp Essential Oil and Extracts

All obtained extracts were tested against a panel of human pathogens comprising nine Gram-positive bacteria, six Gram-negative bacteria, and five fungi (Table 6). Except for *H. pylori* (minimum inhibitory concentration, MIC ≤ 62.5 mg/L), the samples were inactive against the other Gram-negative bacteria. Nevertheless, most Gram-positive strains were sensitive to the treatment with the hemp extracts. The inactivity of hemp against most Gram-negative bacteria has already been proven by other authors [34]. According to the criteria proposed by Kuete and Efferth [40], a sample is considered to have a significant antimicrobial activity if the MIC value is below 100 mg/L. Thus, HEO displayed the most potent antibacterial activity against *S. aureus* (MIC = 62.5 mg/L) and *M. luteus* (MIC = 15.6 mg/L); for the other strains, the MIC values were ≥125 mg/L. In general, the previous literature data also showed a modest activity of hemp EO. For instance, the MIC values ranged from 1200–4700 mg/L for a panel of bacteria comprising *S. aureus*, *B. subtilis*, *M. luteus*, *E. coli*, *P. aeruginosa*, and *K. pneumoniae* [41]. Similar values (MIC = 8000 mg/L) were also reported for hemp EO against different strains of *S. aureus* [42].

HTE showed the highest activity against *S. aureus* (MIC = 0.98 mg/L). For *S. aureus* MRSA, *S. epidermidis*, *M. luteus*, and *B. cereus*, the MIC values displayed by HTE were identical (3.9 mg/L). *Candida* species were not significantly inhibited; however, a strong anti-*Trichophyton* activity (MIC = 31.3 mg/L) was noticed for HTE. Similarly, HSE acted as a potent inhibitor against the growth of *S. aureus* (MIC = 0.98 mg/L), *M. luteus* (MIC = 1.95 mg/L), *E. faecalis* (MIC = 7.8 mg/L), and *B. cereus* (MIC = 1.95 mg/L), and *T. mentagrophytes* (MIC = 31.3 mg/L). The antimicrobial potential of HWE was better than HEO but considerably lower than that exhibited by HTE and HSE (Table 6). This behavior could be linked to the low cannabinoid concentration noticed in HWE compared to HTE and HSE (Table 4).

Our findings are in line with those observed by other authors. For instance, various hemp flower extracts displayed MIC values between 10 and 66 mg/L against *E. coli*, *P. aeruginosa*, *B. subtilis*, and *S. aureus* [43]. Muscara et al. [34] reported MIC values of 39 mg/L against *S. aureus*. On the other hand, Serventi et al. [4] documented for various hemp extracts a potent inhibitory activity against *B. subtilis* (MIC = 1.5–25 mg/L), *S. aureus* (MIC = 12.5–100 mg/L), *P. aeruginosa* (MIC = 25–100 mg/L), and *E. coli* (MIC = 6.2–12.5 mg/L); however, the inhibition of *B. cereus* and *S. typhy* was negligible (MIC > 200 mg/L). Furthermore, the same group of authors also presented the activity of hemp extracts against several dermatophyte strains, with MIC values ranging from 25 to 100 mg/L against *T. mentagrophytes*, *T. tonsurans*, and *T. rubrum* [4].

In an attempt to link the antimicrobial activity of hemp flower extracts to the presence of cannabinoids, the effects of four previously isolated cannabinoids (i.e., CBD, CBG, CBN, and CBC) [44] were subsequently evaluated against the same panel of microbial strains (Table 7). All tested cannabinoids were inactive against Gram-negative bacteria except for *H. pylori* (MIC ≤ 0.98 mg/L for CBG, CBD, and CBN). Furthermore, the four compounds showed no significant inhibitory activity against *Candida* spp. and *Trichophyton* spp. However, the MIC values against the Gram-positive strains indicated promising antibacterial activity, in particular against *S. aureus*, methicillin-resistant *S. aureus* (MRSA), *S. epidermidis*, *M. luteus*, *E. faecalis*, and *B. cereus* (MIC between 0.49 and 15.6 mg/mL). Overall, the following decreasing order of the inhibitory activity against Gram-positive bacteria could be proposed: CBD > CBN > CBG > CBC.

The evidence of antimicrobial activity for CBD has already been proven. For instance, MIC values of 1–4 mg/L were noticed against a diverse array of Gram-positive bacteria, including MRSA, MDR *S. pneumoniae*, *E. faecalis*, and anaerobic *Clostridium difficile* and *Cutibacterium acnes* [45]. CBD also showed different levels of antibacterial activity against Gram-positive bacteria, including susceptible and MDR strains (MIC = 2–4 mg/L for *E. faecium*, *Enterococcus* spp., *Staphylococcus* spp., *M. luteus*, and *Rhodococcus equi*. Furthermore, CBD displayed MIC values of 4–8 mg/L against MRSA, *E. faecalis*, and *L. monocytogenes* [46].

The antimicrobial activity of other minor cannabinoids has been scarcely investigated. For instance, Appendino et al. [5] showed that CBC, Δ^9^-THC, CBN, and CBG could exert antibacterial activity against various MDR *S. aureus* strains, with MIC values between 0.5 and 2 mg/L. In addition, the same cannabinoids also inhibited MRSA, *Streptococcus mutans*, *Streptococcus sanguis*, *Streptococcus sobrinus*, and *Streptococcus salivarius* with MIC below 5 mg/L [47]. Furthermore, *S. aureus*, *S. epidermidis*, and *S. pyogenes* were also shown to be impacted by CBG treatment (MIC = 10–75 μM) [48,49]. Nevertheless, the inhibitory effects of CBG, CBN, and CBC against other human pathogens are reported herein for the first time.

Concerning the possible mechanisms of antimicrobial activity of cannabinoids, it was previously shown that CBD, in combination with bacitracin, caused defects in cell division and irregularities in the cell envelope [46]. The treatment with CBG led to intracellular accumulation of membrane structures, induced membrane hyperpolarization, and decreased membrane fluidity of various bacterial strains [48]. The antibacterial activity of cannabinoids against MRSA was shown to be mediated through the inhibition of biofilm formation, the eradication of pre-formed biofilms and stationary phase cells persistent to antibiotics [47]. Furthermore, CBD proved a potent inhibitor of membrane vesicle release from *E. coli*, altering cell communication [50]. Nevertheless, the antimicrobial mechanisms of CBD and minor cannabinoids remain elusive, requiring subsequent investigations.

## 3. Materials and Methods

### 3.1. Materials

Alkane standard solution (C8–C20, ~40 mg/L each, in hexane), fatty acid methyl ester (FAME) mix (C8–C24), Mueller–Hinton (MH) broth, bisabolol (≥93%), caryophyllene (≥98%), myrcene (≥98%), linalool (≥99%), tridecanoic acid (≥98%), hydrochloric acid (≥37%), and glucose (≥99%) were acquired from Merck KGaA (Darmstadt, Germany). Hexane (≥95%), ethanol (≥99%), methanol (≥99%), acetonitrile (≥99%), and water (≥99%) were from VWR Chemicals (Ismaning, Germany). Caryophyllene oxide (≥95%) was bought from Thermo Scientific (Olching, Germany), whereas humulene (≥97%) was purchased from Biomol (Hamburg, Germany). The certified reference materials of 13 cannabinoids containing cannabidivarinic acid (CBDVA), cannabidivarin (CBDV), cannabigerolic acid (CBGA), cannabigerol (CBG), cannabidiol (CBD), tetrahydrocannabivarin (THCV), cannabinol (CBN), Δ^9^-tetrahydrocannabinol (Δ^9^-tTHC), Δ^8^-tetrahydrocannabinol (Δ^8^-THC), cannabicyclol (CBL), cannabichromene (CBC), and Δ^9^-tetrahydrocannabinolic acid (Δ^9^-THCA) was supplied from LGC Standards (Kielpin, Poland). The dried *Cannabis sativa* (hemp) flowers (cv. Futura 75) were provided by Hempartis GmbH (Malsch, Germany).

### 3.2. Preparation of Essential Oil and Solvent Extracts

The powdered hemp flowers (50 g) were subjected to hydrodistillation on a Clevenger-type apparatus with 500 mL water for 3 h. At the end of the hydrodistillation process, the amount of essential oil (HEO) was measured using the apparatus scale (in mL), collected, and dried over anhydrous sulfate. The water in the flask was filtered, and 250 mL were freeze-dried to afford the residual water extract (HWE). The solid plant material residue (spent hemp flowers) was dried in an oven at 40 °C for 48 h, and 7.5 g were extracted with hexane (75 mL) in an ultrasound bath for 30 min, for 3 repeated cycles, each time with the same volume of fresh solvent. After filtration, the solvent was evaporated under reduced pressure, yielding the spent extract (HSE). For comparison purposes, the unprocessed powdered hemp flowers were extracted with the same solvent under the same conditions, affording the total (unprocessed material) extract (HTE). For HEO, the yield was calculated with the following formula:

$$\%yield=\frac{volume\;of\;the\;obtained\;oil(mL)}{mass\;of\;the\;hemp\;flowers(g)}\times 100$$

For the spent extract (HSE), the yield was determined as follows:

$$\%yield=\frac{mass\;of\;the\;obtained\;extract\;after\;drying(g)}{mass\;of\;the\;spent\;hemp\;flowers(g)}\times 100$$

For the total extract (HTE), the yield was calculated with the formula:

$$\%yield=\frac{mass\;of\;the\;obtained\;extract\;after\;drying(g)}{mass\;of\;the\;hemp\;flowers(g)}\times 100$$

For HWE, the yield was determined using the formula:

$$\%yield=\frac{m_{1}\left(g\right)}{m_{2}(g)}\times \frac{V_{1}(mL)}{V_{2}(mL)}\times 100$$

where $m_{1}$ is the mass of the obtained water extract after freeze-drying, $m_{2}$ is the mass of the hemp flowers, $V_{1}$ is the volume of the water introduced in the hydrodistillation, and $V_{2}$ is the volume of the freeze-dried water.

All extractions were performed in triplicate, with the extraction yields provided in Table 1.

### 3.3. Analytical Methods

#### 3.3.1. Terpene Profile of Hemp Essential Oil (GC–MS)

The terpene profile of HEO was assessed by GC–MS performed on a TRACE GC Ultra instrument (Thermo Fischer, Waltham, MA, USA). The chromatographic separations were conducted on a Zebron ZB-5MS (30 m × 0.25 mm, 0.25 μm) column (Phenomenex, Torrance, CA, USA), with helium as the carrier gas at a flow rate of 1.43 mL/min. The injection temperature was 250 °C; 1 μL was injected with a split ratio of 50:1. The column temperature was initially held at 60 °C for 4 min, then increased to 280 °C at a rate of 10 °C/min, and maintained at 280 °C for 5 min. The MS parameters included: transfer line temperature, 320 °C; source temperature, 230 °C; ionization energy, 70 eV. The linear retention indices (LRI) were determined for all compounds in the chromatograms using a standard mixture of alkanes ranging from 8 to 20 carbon atoms. Peak identification was achieved by referencing the NIST 11 Mass Spectra Library and comparing the calculated LRI with those found in the relevant literature. All analyses were performed in triplicate.

#### 3.3.2. Cannabinoid Profile and Composition

The cannabinoid profile of solvent extracts (HTE, HSE, and HWE) was assessed by LC–HRMS/MS. An Agilent 1200 HPLC (Agilent Technologies, PaloAlto, CA, USA), comprising an auto-sampler (G1329B), a binary pump (G1312C), and a column thermostat (G1316A), was used. The instrument was connected to an accurate-mass quadrupole time-of-flight (QTOF) MS/MS system from the Agilent 6530B series via a dual electrospray ionization (ESI) interface. The chromatographic separations were conducted on a Gemini C18 (100 mm × 2 mm, 3 μm) column (Phenomenex, Torrance, CA, USA) operated at 25 °C. The mobile phase comprised (A) water and (B) acetonitrile, both containing 0.1% formic acid. The phases were delivered at a flow rate of 0.2 mL/min, in the following gradient: 50–55% B from 0 to 12 min, 55–58% from 12 to 25 min, 58–75% B from 25 to 30 min, 75–90% B from 30 to 45 min, 90% B from 45 to 50 min. The sample injection volume was 10 μL. The detection was carried out in positive electrospray ionization mode, with the spectra recorded in the *m*/*z* 100–1000 Da range. The ion source parameters were as follows: drying gas (nitrogen) flow rate, 12 L/min; heated capillary temperature, 300 °C; nebulizer pressure, 35 psi; sheath gas temperature, 275 °C; sheath gas flow rate, 12 L/min; capillary voltage, 4000 V; nozzle voltage, 2000 V; fragmentor, 110 V; skimmer, 65 V; octupole radiofrequency peak voltage, 750 V. The MS/MS spectra were generated by automated fragmentation at a fixed collision energy of 30 V.

The cannabinoid concentration of solvent extracts (HTE, HSE, and HWE) was assessed by LC–DAD on a Shimadzu HPLC (Tokyo, Japan) containing a binary pump (LC-20AD), autosampler (SIL-20A), degasser (DGU-20A), and UV/VIS detector (SPD-M20A). The chromatographic separations were conducted on a Zorbax XDB-C18 (150 mm × 4.6 mm, 3.5 μm) column (Agilent Technologies, Palo Alto, CA, USA) at 30 °C. The mobile phase comprised (A) water and (B) acetonitrile, both containing 0.1% formic acid. The phases were delivered at a flow rate of 1.5 mL/min in the following gradient: 72% B for 4 min, 75–80% B from 4.01 to 11 min, and 90% B from 11.01 to 12 min. The sample injection volume was 10 μL, with the chromatograms recorded at 228 nm. The concentrations of cannabinoids were assessed using the calibration curves of the corresponding standards. All analyses were performed in triplicate.

#### 3.3.3. Fatty Acid Composition of Hemp Extracts (GC–MS)

The fatty acid composition of solvent extracts (HTE, HSE, and HWE) was assessed by GC–MS on an Agilent 6890 GC (Agilent Technologies, PaloAlto, CA, USA) coupled to a mass selective detector (MSD). The chromatographic separations were conducted on a Zebron Rtx-Wax (30 m × 0.25 mm, 0.25 μm) column (Restek, Centre County, PA, USA), with helium as the carrier gas at a flow rate of 1 mL/min. The injection temperature was 200 °C; 1 μL was injected with a split ratio of 7.5:1. The column temperature was initially held at 80 °C for 2 min, then increased to 180 °C at a rate of 7 °C/min, maintained at 180 °C for 10 min; then ramped up to 230 °C at a rate of 1 °C/min, and finally held at 230 °C for 10 min. The MS parameters included: transfer line temperature, 230 °C; source temperature, 230 °C; ionization energy, 70 eV. Before analyses, the samples (~20 mg) were dissolved in 1 mL hexane. Then, 0.2 mL of the obtained solutions were incubated at 90 °C for 1 h in the presence of 6 mL of methanol/hydrochloric acid (11:1, *v*/*v*) and 0.1 mL tridecanoic acid (1 mg/mL in hexane) as internal standard. Next, 1.7 mL hexane and 2 mL water were added; after shaking and phase separation, 1 mL of the upper phase was analyzed by GC–MS. The fatty acid content in the samples was expressed based on a calibration curve performed with the FAME mixture. All analyses were performed in triplicate.

### 3.4. Antimicrobial Assays

The antimicrobial assays were performed using the microdilution method, according to the European Committee on Antimicrobial Susceptibility Testing [51,52]. The antimicrobial activity was evaluated against Gram-positive bacteria (*Staphylococcus aureus* ATCC 25923, *Staphylococcus aureus* ATCC BAA-1707, *Staphylococcus epidermidis* ATCC 12228, *Micrococcus luteus* ATCC 10240, *Enterococcus faecalis* ATCC 29212, *Bacillus cereus* ATCC 10876, *Streptococcus pneumoniae* ATCC 49619, *Streptococcus pyogenes* ATCC 19615, *Streptococcus mutans* ATCC 25175), Gram-negative bacteria (*Helicobacter pylori* ATCC 43504, *Salmonella* Typhimurium ATCC 14028, *Escherichia coli* ATCC 25922, *Proteus mirabilis* ATCC 12453, *Klebsiella pneumoniae* ATCC 13883, *Pseudomonas aeruginosa* ATCC 9027), and fungi (*Candida albicans* ATCC 102231, *Candida parapsilosis* ATCC 22019, *Candida glabrata* ATCC 2091, *Trichophyton rubrum* ATCC 28188, *Trichophyton mentagrophytes* ATCC 9533). Serial double dilutions of samples were prepared in MH broth or RPMI 1640 medium 2% glucose buffered with 0.165 M MOPS and supplemented with chloramphenicol 50 mg/L and cycloheximide 300 mg/L, for non-fastidious bacteria and fungi, respectively. The sterile 96-well flat-bottom polystyrene microtitrate plates (Nunc, Denmark) were prepared by dispensing 100 µL of the appropriate dilution of the tested extracts in broth medium per well by serial two-fold dilutions, to obtain the final concentrations of the tested extracts ranging from 2000 to 0.25 mg/L for bacteria and yeasts. The inocula were prepared with fresh microbial cultures in sterile 0.85% NaCl to match the turbidity of 0.5 McFarland standard, and were added to wells to obtain the final density of 5 × 10^5^ colony forming units (CFU)/mL for bacteria, 5 × 10^4^ CFU/mL for yeasts and 5 × 10^5^ CFU/mL for dermatophytes. After incubation (non-fastidious bacteria and yeasts—35 °C for 24 h and dermatophytes—28 °C for 5 days), the growth of microorganisms was measured spectrophotometrically at 600 nm (BioTEK ELx808, BioTek Instruments, Inc., Winooski, VT, USA). The minimum inhibitory concentration (MIC) for *H. pylori* ATCC 43504 was determined using a two-fold microdilution method in MH broth with 7% of lysed horse blood at an extract concentration ranging from 2000 to 0.25 mg/L with a *H. pylori* suspension of 3 McFarland standard diluted 100 times (9 × 10^6^ CFU/mL). After incubation at 35 °C for 72 h under microaerophilic conditions (5% O_2_, 15% CO_2_, and 80% N_2_), the growth of *H. pylori* was visualized with the addition of 10 µL of 0.04% resazurin. The MIC endpoint was recorded after 4 h incubation as the lowest concentration of extract that completely inhibits bacterial growth. An appropriate DMSO control (at a final concentration of 10%), a positive control (containing inoculum without the tested extracts), and a negative control (containing the tested extracts without inoculum) were included on each microplate. The MIC was determined and reported for each sample and strain. Vancomycin, ciprofloxacin, ofloxacin, and nystatin/terbinafine were used as the standard reference drugs. All experiments were performed in triplicate.

### 3.5. Statistical Analysis

Data are provided as mean ± standard deviation of three repeated experiments; ANOVA with Tukey’s post hoc test was conducted; *p* < 0.05 was considered statistically significant.

## 4. Conclusions

The seminal findings provided by the phytochemical analysis are as follows: (i) 15 monoterpenes and 36 sesquiterpenes were identified in the EO, with sesquiterpenes accounting for ~85% of total peak area; (ii) a total of 7 acidic cannabinoids and 14 neutral derivatives were annotated in the post-distillation by-products, with CBD as the dominant compound (up to 16 wt.%); (iii) linoleic and linolenic acid were the representative fatty acids in the solvent extracts (up to 2 wt.%); (iv) the spent extracts displayed cannabinoid levels around 2–3 times higher than the unprocessed flower solvent extracts. Concerning the biological study, the hemp EO and extracts demonstrated potent antimicrobial activity (MIC < 62.5 mg/L) against Gram-positive bacteria (e.g., *S. aureus*, *S. epidermidis*, *M. luteus*, *E. faecalis*, *B. cereus*, *S. pneumoniae*), *H. pylori*, and *Trichophyton* spp. In addition, when CBD, CBG, CBN, and CBC were individually tested against the same panel of microorganisms, MIC values ranging from 0.49 and 15.6 mg/mL against Gram-positive bacteria were retrieved. The inhibitory activity generally decreased in the following order: CBD > CBN > CBG > CBC. For some cannabinoids, the antimicrobial properties against certain microbial strains were proven for the first time in the current study.

Considering that the hemp flower essential oil industry generates significant amounts of unused biomass rich in cannabinoids, the strategy implemented in the current work could afford high-added-value by-products within the hemp production chain, contributing to the principles of the circular economy and sustainability. Altogether, this work can open promising avenues for utilizing cannabinoid-rich materials obtained during hemp flower processing in functional foods or cosmeceutical and pharmaceutical products with antimicrobial properties.

## Figures and Tables

**Table 1:** Extraction yields of hemp essential oil and extracts.

Extract	Code	Unit	Yield
Hemp flower essential oil	HEO	mL/100 g	0.7 ± 0.1 ^a^
Total (unprocessed) hemp flower extract	HTE	g/100 g	7.3 ± 0.3 ^b^
Spent (processed) hemp flower extract	HSE		8.2 ± 0.4 ^c^
Hydrodistillation water extract	HWE		6.4 ± 0.2 ^d^

Data are reported as mean ± SD of three experiments; different letters within columns indicate significant differences (*p* < 0.05).

**Table 2:** Terpene profile of hemp essential oil.

No.	Compound	LRI ^a^	LRI ^b^	Relative Abundance (%) ^c^
1	*α*-Pinene	936	936	2.80 ± 0.04
2	*β*-Pinene	980	978	0.73 ± 0.00
3	*β*-Myrcene *	989	989	1.09 ± 0.02
4	Limonene *	1032	1033	0.24 ± 0.00
5	Eugenol	1035	1034	0.41 ± 0.01
6	Linalool *	1099	1103	0.55 ± 0.01
7	Fenchyl alcohol	1124	1117	0.32 ± 0.01
8	*cis*-*p*-Menth-2-en-1-ol	1131	1121	0.24 ± 0.00
9	*trans*-*p*-Menth-2-en-1-ol	1140	1140	0.32 ± 0.01
10	Camphor	1150	1148	0.12 ± 0.00
11	Borneol	1178	1177	0.24 ± 0.01
12	Terpinen-4-ol	1185	1180	0.13 ± 0.01
13	*p*-Cymen-8-ol	1199	1185	0.49 ± 0.02
14	α-Terpineol	1198	1198	0.46 ± 0.05
15	Copaene	1376	1378	0.20 ± 0.01
16	(*Z*)-*β*-Caryophyllene	1414	1409	0.20 ± 0.01
17	*γ*-Elemene	1418	1430	0.33 ± 0.01
18	(*E*)-*β*-Caryophyllene *	**1431**	**1428**	**17.17 ± 0.24**
19	*α*-Bergamotene	1438	1435	1.87 ± 0.03
20	*allo*-Aromadendrene	1443	1444	0.49 ± 0.01
21	(*E*)-*β*-Farnesene	1453	1456	1.59 ± 0.03
22	*α*-Humulene *	**1467**	**1458**	**6.12 ± 0.53**
23	*γ*-Muurolene	1472	1476	0.25 ± 0.02
24	*α*-Guaiene	1482	1499	0.34 ± 0.06
25	Selina-4(14),7(11)-diene	1485	1497	0.60 ± 0.08
26	*β*-Selinene	1495	1486	0.80 ± 0.03
27	*α*-Selinene	1501	1497	2.85 ± 0.01
28	***β*-Bisabolene**	**1511**	**1509**	**5.14 ± 0.05**
29	*δ*-Cadinene	1520	1519	0.73 ± 0.02
30	*γ*-Patchoulene	1525	1522	0.47 ± 0.01
31	*β*-Sesquiphellandrene	1530	1525	1.06 ± 0.03
32	*α*-Cadinene	1544	1530	1.48 ± 0.03
33	Selina-3,7(11)-diene	1549	1542	0.76 ± 0.02
34	Selina-4(15),7(11)-diene	1554	1532	0.38 ± 0.01
35	Germancrene B	1563	1157	1.48 ± 0.03
36	(*E*)-Nerolidol	1568	1560	0.15 ± 0.01
37	Spathulenol	1575	1573	0.42 ± 0.02
38	*β*-Caryophyllene oxide *	**1597**	**1581**	**8.22 ± 0.13**
39	Aromadendrene oxide	1606	1650	2.87 ± 0.04
40	*cis*-(*Z*)-*α*-Bisabolene epoxide	1613	1619	0.85 ± 0.01
41	Humulene epoxide	1619	1610	0.41 ± 0.02
42	Cubenol	1625	1614	2.70 ± 0.04
43	*γ*-Eudesmol	1638	1642	4.02 ± 0.06
44	Caryophylla-4(12),8(13)-dien-5*α*-ol	1648	1640	2.29 ± 0.02
45	Caryophylla-4(12),8(13)-dien-5*β*-ol	1651	1634	1.70 ± 0.08
46	*α*-Cadinol	1657	1647	0.53 ± 0.03
47	***β*-Eudesmol**	**1670**	**1651**	**5.79 ± 0.05**
48	*epi*-*α*-Bisabolol	1677	1682	3.49 ± 0.07
49	α-Bisabolol *	**1692**	**1685**	**8.20 ± 0.12**
50	Cannabidiol *	–	–	1.75 ± 0.03
	**Total**			**95.82 ± 1.35**
	Hydrocarbon monoterpenes			4.86 ± 0.02
	Oxygenated monoterpenes			3.33 ± 0.10
	Hydrocarbon sesquiterpenes			44.33 ± 0.62
	Oxygenated sesquiterpenes			41.61 ± 0.64

^a^ Linear retention index on ZB-5MS column; ^b^ Linear retention indices on ZB-5MS column or equivalent columns according to the NIST library (webbook.nist.gov, accessed on 2 February 2024); ^c^ Expressed as the mean percentage area extracted from the GC–MS chromatograms of three repeated analyses; in bold are the major terpenes; * confirmed by standard.

**Table 3:** Cannabinoid profile of hemp extracts.

No	Proposed Identity	T_R_ (min)	HRMS (*m*/*z*)	MF	HRMS/MS (*m*/*z*)	Sample	Ref.
1	Cannabichromanonic acid	4.1	377.1974	C_21_H_28_O_6_	301.1463, 283.1369, 273.1480, 255.1386, 245.1524	HTE, HSE	[29]
2	Cannabielsoic acid	5.2	375.2153	C_22_H_30_O_5_	357.2051, 339.1941, 297.1521, 275.1269, 245.1509, 233.1140, 219.0995, 207.1004	HTE, HSE, HWE	[29,30]
3	Cannabinodiolic acid	5.9	355.1892	C_22_H_26_O_4_	323.1605, 313.1776, 299.1625, 273.1465, 253.0850, 239.1048, 225.0896, 211.0977, 197.0944, 187.0741	HTE, HSE, HWE	[29,30,32]
4	Cannabitriolic acid	7.0	391.2091	C_22_H_30_O_6_	301.1454, 283.1310, 273.1460, 255.1353, 245.1510, 235.0933, 217.0834	HTE, HSE, HWE	[29]
5	Cannabidivarinic acid *	8.2	331.1893	C_20_H_26_O_4_	313,1781, 295.0933, 273.1487, 255.1346, 215.1534, 193.1198, 173.0941, 145.0996, 141.0894	HTE, HSE, HWE	[29,30,32]
6	Cannabitriol	10.2	347.2195	C_21_H_30_O_4_	291.1574, 271.1515, 245.1521, 231.1353, 217.1195, 207.1053, 201.0889, 193.1195	HTE, HSE, HWE	[29,30]
7	Cannabinodiol	11.6	311.1995	C_21_H_26_O_2_	281.1500, 231.1358, 225.0907, 217, 1210, 213.0901, 199.0744, 193.1200, 173.0945, 165.0893, 145.0978	HTE, HSE, HWE	[29,30]
8	Epoxycannabigerol	12.7	333.2339	C_21_H_32_O_3_	273.1846, 259.1678, 247.1653, 193.1203, 177.1216, 135.1143, 123.0422	HTE, HSE, HWE	[29,30]
9	Cannabidivarin *	13.9	287.2002	C_19_H_26_O_2_	231.1375, 219.136, 203.1074, 189.0911, 179.059, 165.0907, 123.0439	HTE, HSE, HWE	[29,30,31]
10	Cannabielsoin	15.3	331.2249	C_21_H_30_O_3_	271.1670, 231.1360, 193.1204, 135.0426, 109.1000	HTE, HSE, HWE	[29,30,31]
11	Δ^9^/Δ^8^-Tetrahydrocannabinolic acid	16.5	345.2059	C_21_H_28_O_4_	327.1949, 297.1498, 285.1515, 268.1521, 229.0866, 211.0762, 197.0623	HTE, HSE, HWE	[29,30,31,32]
12	Hydroxycannabinol	18.2	327.1978	C_21_H_26_O_3_	313.2111, 287.1663, 271.1692, 259.1686, 247.1362, 231.1355, 211.0768, 201.0899, 193.1208	HTE, HSE, HWE	[30]
13	Cannabicoumaronic acid	20.6	373.1988	C_22_H_28_O_5_	299.1685, 273.1462, 233.1158, 193.1122, 183.0999, 147.0787, 127.0376	HTE, HSE	[29]
14	Cannabidiolic acid *	21.5	359.2201	C_22_H_30_O_4_	341.2096, 299.1638, 285.1469, 273.1474, 261.1467, 233.1159, 219.1001	HTE, HSE, HWE	[29,30,32]
15	Cannabidiol *	22.6	315.2311	C_21_H_30_O_2_	273.1825, 259.1677, 247.1676, 231.1373, 217.1209, 207.21363, 193.1209, 177.1189, 165.0901, 151.0744, 137.0588, 135.1155, 123.0424, 107.0844	HTE, HSE, HWE	[29,30,31,32]
16	Cannabinol *	26.1	311.2001	C_21_H_26_O_2_	273.1866, 259.1670, 241.1544, 231.1374, 217.1217, 193.1213, 135.1154, 123.0433, 107.0848	HTE, HSE, HWE	[29,30,31,32]
17	Dehydrocannabifuran	29.7	309.1829	C_21_H_24_O_2_	294.1607, 281.1576, 253.1205, 238.0974, 235.1106, 225.1198	HTE, HSE	[29,30]
18	Δ^9^/Δ^8^-Tetrahydrocannabinol *	32.2	315.2304	C_21_H_30_O_2_	273.1844, 259.1680, 247.1673, 231.1377, 217.125, 207.1355, 193.1211, 135.1158, 123.0433, 107.847	HTE, HSE, HWE	[29,30,31,32]
19	Cannabicoumaronone	33.3	329.2095	C_21_H_28_O_3_	287.1522, 273.1444, 259.1655, 247.1320, 229.0827, 209.1138, 153.0543	HTE, HSE	[30]
20	Cannabicyclol *	38.1	315.2308	C_21_H_30_O_2_	273.1792, 259.1681, 247.1697, 233.1519, 217.1224, 207.1359, 193.1208, 177.1217, 135.1160, 123.0431	HTE, HSE, HWE	[29,30,31]
21	Cannabichromene *	39.1	315.2316	C_21_H_30_O_2_	259.1659, 247.1670, 231.1372, 217.1351, 193.1193, 177.1175, 165.0884, 151.0731, 135.1141, 123.0420	HTE, HSE	[29,30,31]

* confirmed by standard.

**Table 4:** Cannabinoid concentration of the hemp flower extracts.

Sample	HTE	HSE	HWE
Cannabinoid		wt.%	
Cannabidivarinic acid	–	–	–
Cannabidivarin	0.35 ± 0.01 ^a^	0.21 ± 0.05 ^b^	0.02 ± 0.00 ^c^
Cannabidiolic acid	1.11 ± 0.01 ^a^	0.06 ± 00 ^b^	0.01 ± 0.00 ^c^
Cannabigerolic acid	–	–	–
Cannabigerol	0.07 ± 0.00 ^a^	0.38 ± 0.00 ^b^	0.01 ± 0.00 ^c^
Cannabidiol	7.02 ± 0.02 ^a^	15.93 ± 0.02 ^b^	0.76 ± 0.00 ^c^
Tetrahydrocannabivarin	0.11 ± 0.00 ^a^	0.02 ± 0.00 ^b^	-
Cannabinol	0.54 ± 0.02 ^a^	0.55 ± 0.01 ^a^	0.04 ± 0.00 ^b^
Δ^9^-Tetrahydrocannabinol	0.25 ± 0.02 ^a^	0.29 ± 0.01 ^b^	0.01 ± 0.00 ^c^
Δ^8^-Tetrahydrocannabinol	0.23 ± 0.01 ^a^	0.02 ± 0.00 ^b^	-
Cannabicyclol	0.02 ± 0.00 ^a^	0.06 ± 0.00 ^b^	0.01 ± 0.00 ^c^
Cannabichromene	0.02 ± 0.00 ^a^	0.02 ± 0.00 ^a^	-
Δ^9^-Tetrahydrocannabinolic acid	0.03 ± 0.00 ^a^	0.02 ± 0.00 ^b^	-

Data are expressed as mean ± SD of three repeated analyses; sample codes as in Table 1; different letters within rows indicate significant differences (*p* < 0.05).

**Table 5:** Quantification of fatty acid derivatives in the hemp flower extracts.

Sample	HTE	HSE	HWE
Fatty acid		wt.%	
Myristic acid (C14:0)	0.09 ± 0.00 ^a^	0.17 ± 0.01 ^b^	-
Palmitic acid (C16:0)	0.88 ± 0.03 ^a^	0.86 ± 0.01 ^a^	0.03 ± 0.01 ^b^
Stearic acid (C18:0)	0.09 ± 0.00 ^a^	0.10 ± 0.00 ^b^	-
Oleic acid (C18:1)	0.29 ± 0.01 ^a^	0.36 ± 0.01 ^b^	-
Linoleic acid (C18:2)	1.52 ± 0.08 ^a^	1.75 ± 0.07 ^b^	0.03 ± 0.01 ^c^
Linolenic acid (C18:3)	1.39 ± 0.08 ^a^	1.26 ± 0.09 ^a^	0.14 ± 0.00 ^b^
Arachidic acid (C20:1)	0.58 ± 0.02 ^a^	0.89 ± 0.03 ^b^	-
Behenic acid (C22:0)	0.13 ± 0.01 ^a^	0.20 ± 0.02 ^b^	-

Data are expressed as mean ± SD of three repeated analyses; sample codes as in Table 1; different letters within rows indicate significant differences (*p* < 0.05).

**Table 6:** Antimicrobial activity of hemp essential oil and extracts.

Extract	HEO	HSE	HTE	HWE	Antibiotics
Microorganism	MIC [mg/L]				
**Gram-positive bacteria**					
*Staphylococcus aureus*	62.5 ^a^	0.98 ^b^	0.98 ^b^	31.3 ^c^	0.98 ^#^
*Staphylococcus aureus* *	1000 ^a^	3.9 ^b^	3.9 ^b^	125 ^c^	0.98 ^#^
*Staphylococcus epidermidis*	1000 ^a^	3.9 ^b^	3.9 ^b^	125 ^c^	0.12 ^#^
*Micrococcus luteus*	15.6 ^a^	1.95 ^b^	3.9 ^c^	62.5 ^d^	1.95 ^#^
*Enterococcus faecalis*	2000 ^a^	7.8 ^b^	7.8 ^b^	250 ^c^	0.24 ^#^
*Bacillus cereus*	500 ^a^	1.95 ^b^	3.9 ^c^	62.5 ^d^	0.98 ^#^
*Streptococcus pneumoniae*	125 ^a^	31.3 ^b^	62.5 ^c^	62.5 ^c^	0.24 ^#^
*Streptococcus pyogenes*	250 ^a^	31.3 ^b^	62.5 ^c^	1000 ^d^	0.24 ^#^
*Streptococcus mutans*	250 ^a^	125 ^b^	125 ^b^	2000 ^c^	0.98 ^#^
**Gram-negative bacteria**					
*Helicobacter pylori*	15.6 ^a^	7.8 ^b^	7.8 ^b^	62.5 ^c^	0.98 ^$^
*Salmonella* Typhimurium	>2000	>2000	>2000	>2000	0.06 ^§^
*Escherichia coli*	>2000	>2000	>2000	>2000	0.02 ^§^
*Proteus mirabilis*	>2000	>2000	>2000	>2000	0.03 ^§^
*Klebsiella pneumoniae*	>2000	>2000	>2000	>2000	0.12 ^§^
*Pseudomonas aeruginosa*	>2000	>2000	>2000	>2000	0.49 ^§^
**Yeasts**					
*Candida albicans*	1000 ^a^	2000 ^b^	250 ^c^	>2000	0.49 ^ß^
*Candida parapsilosis*	500 ^a^	2000 ^b^	250 ^c^	2000 ^b^	0.24 ^ß^
*Candida glabrata*	500 ^a^	>2000	250 ^b^	>2000	0.24 ^ß^
*Trichophyton rubrum*	125 ^a^	125 ^a^	31.3 ^b^	1000 ^c^	0.0049 ^&^
*Trichophyton mentagrophytes*	125 ^a^	31.3 ^b^	31.3 ^b^	250 ^c^	0.0012 ^&^

^#^ vancomycin; ^$^ ofloxacin; ^§^ ciprofloxacin; ^ß^ nystatin; ^&^ terbinafine; sample codes as in Table 1; * methicillin-resistant strain; different letters within rows indicate significant differences (*p* < 0.05); MIC, minimum inhibitory concentration.

**Table 7:** Antimicrobial activity of selected cannabinoids.

Compound	CBG	CBD	CBN	CBC	Antibiotics
Microorganism	MIC [mg/L]				
**Gram-positive bacteria**					
*Staphylococcus aureus*	1.95 ^a^	0.49 ^b^	1.95 ^a^	3.95 ^c^	0.98 ^#^
*Staphylococcus aureus* *	1.95 ^a^	1.95 ^a^	1.95 ^a^	15.6 ^b^	0.98 ^#^
*Staphylococcus epidermidis*	1.95 ^a^	1.95 ^a^	1.95 ^a^	1.95 ^a^	0.12 ^#^
*Micrococcus luteus*	1.95 ^a^	0.98 ^b^	0.98 ^b^	0.98 ^b^	1.95 ^#^
*Enterococcus faecalis*	3.9 ^a^	1.95 ^b^	1.95 ^b^	0.98 ^c^	0.24 ^#^
*Bacillus cereus*	1.95 ^a^	0.98 ^b^	1.95 ^a^	7.8 ^c^	0.98 ^#^
*Streptococcus pneumoniae*	31.3 ^a^	31.3 ^a^	62.5 ^b^	62.5 ^b^	0.24 ^#^
*Streptococcus pyogenes*	31.3 ^a^	31.3 ^a^	62.5 ^b^	62.5 ^b^	0.24 ^#^
*Streptococcus mutans*	31.3 ^a^	31.3 ^a^	62.5 ^b^	62.5 ^b^	0.98 ^#^
**Gram-negative bacteria**					
*Helicobacter pylori*	0.49 ^a^	0.98 ^b^	0.98 ^b^	15.6 ^c^	0.98 ^$^
*Salmonella* Typhimurium	>2000	>2000	>2000	>2000	0.06 ^§^
*Escherichia coli*	>2000	>2000	>2000	>2000	0.02 ^§^
*Proteus mirabilis*	>2000	>2000	>2000	>2000	0.03 ^§^
*Klebsiella pneumoniae*	>2000	>2000	>2000	>2000	0.12 ^§^
*Pseudomonas aeruginosa*	>2000	>2000	>2000	>2000	0.49 ^§^
**Yeasts**					
*Candida albicans*	500 ^a^	500 ^a^	1000 ^b^	500 ^a^	0.49 ^ß^
*Candida parapsilosis*	1000 ^a^	1000 ^a^	1000 ^a^	500 ^b^	0.24 ^ß^
*Candida glabrata*	250 ^a^	1000 ^b^	1000 ^b^	1000 ^b^	0.24 ^ß^
*Trichophyton rubrum*	1000 ^a^	1000 ^a^	1000 ^a^	1000 ^a^	0.0049 ^&^
*Trichophyton mentagrophytes*	1000 ^a^	1000 ^a^	1000 ^a^	1000 ^a^	0.0012 ^&^

^#^ vancomycin; ^$^ ofloxacin; ^§^ ciprofloxacin; ^ß^ nystatin; ^&^ terbinafine; * methicillin-resistant strain; different letters within rows indicate significant differences (*p* < 0.05); CBG, cannabigerol; CBD, cannabidiol; CBN, cannabinol; CBC, cannabichromene; MIC, minimum inhibitory concentration.

## Data Availability

Data are contained within the article.
